# Multilevel model to assess sources of variation in follicular growth close to the time of ovulation in women with normal fertility: a multicenter observational study

**DOI:** 10.1186/1477-7827-6-61

**Published:** 2008-12-10

**Authors:** Rafael T Mikolajczyk, Joseph B Stanford, René Ecochard

**Affiliations:** 1 Department of Public Health Medicine, School of Public Health, University of Bielefeld, Bielefeld, Germany; 2 Department of Family and Preventive Medicine, University of Utah, Utah, USA; 3 Hospices Civils de Lyon, Service de Biostatistique, Lyon, France; 4 CNRS, UMR 5558 Equipe Biostatistique-Santé, Villeurbanne, France; 5 Université Lyon 1, UMR 5558 Laboratoire Biostatistique-Santé, Villeurbanne, France

## Abstract

**Background:**

To assess the amount of variability in ovarian follicular growth rate and maximum follicular diameter related to different centers, women and cycles of the same women in a multicenter observational study of follicular growth.

**Methods:**

Secondary analysis of a prospective cohort study from eight centers in Europe. There were 533 ultrasound examinations in 282 cycles of 107 women with normal fertility. A random effects model with center, woman and cycle as hierarchical units of variation was used to analyze mean follicular diameter on days preceding ovulation.

**Results:**

Follicular growth did not differ by center. There was homogenous growth across women and cycles, and the maximum follicular diameter before ovulation varied substantially across cycles but not across women. Many (about 40%) women had small maximum follicular diameter on the day before ovulation (<19 mm). Pre-ovulatory cycle length was not related to maximum follicular diameter.

**Conclusion:**

In normal fecundity, there is a substantial variation in maximum follicular diameter from cycle to cycle based on variation in the duration of follicular development, but the variation could not be explained by different characteristics of different women. Explanation of variation in follicular growth has to be found on the cycle level.

## Background

A number of studies have assessed the accuracy of ultrasound to monitor ovarian follicular growth and predict the timing of ovulation. Earlier studies focused solely on describing the growth curve in terms of mean values of follicular diameter on different days. Some of these studies analyzed the growth relative to the beginning of the growth phase [1,2], and others relative to ovulation, either as demonstrated by ultrasound or by an additional marker of ovulation [3-12]. Most researchers have suggested a linear growth model (with respect to the follicular diameter), although Queenan *et al*. [7] found some evidence for exponential growth. One of the larger published studies demonstrated a particularly low predictive value of follicular diameter for the timing of ovulation [10]. In general, wide variation has been documented in follicular diameter prior to ovulation. Such variability in follicular growth may be attributable to the population that the woman comes from, the woman's personal characteristics, or factors specific to a particular menstrual cycle. However, no studies have formally assessed where the variability in follicular growth arises: between women from different populations, between individual women, or within the same woman's cycles. Making such distinctions may suggest different possible determinants of follicular development. For example, finding that there is variation between different women but little variation between cycles of the same woman would suggest that the main source of variation is characteristics of different women. In contrast, finding that there is little variation between women but a substantial variation between the cycles of the same woman would suggest that the main source of variation is short-term exposures or characteristics of each cycle. Finally, the amount of variation between different populations of women would suggest possible population-based characteristics influencing follicular growth. The aim of our analysis was to assess the sources of variation in follicular growth trajectories in natural (non-stimulated) cycles of women with normal fecundity, assessing simultaneously the differences due to population-level, woman-level, and cycle-level influences, using multi-level statistical modeling.

## Methods

### Sample

We utilized data from a study that assessed multiple peri-ovulatory events in women of normal fertility including serial ovarian ultrasound with follicle measurement. A previous analysis of this study reported mean follicular diameter on the days preceding ovulation [13], but did not analyze the growth trajectories. The data set consists of 327 cycles in 107 women who were not taking hormonal medication and had no history that would suggest subfecundity. The subjects were recruited from 8 different centers in 4 countries in Europe. The inclusion criteria were: ostensibly healthy menstruating women; aged 18 to 45 inclusive; with previous menstrual cycle lengths of 24 to 34 days inclusive; and experience in natural family planning methods (basal body temperature and signs of cervical mucus). Women with frequent anovulatory cycles, on any hormonal treatment, with known disturbances of follicular development or with a history of infertility were excluded. The subjects of the study were between 20 and 45 years old, with median of 33.6 years and 50% of the sample between 29 and 39 years. More than 60% were of proven fertility, having at least one child before the study.

### Measurements

On each of the cycle days, women recorded their basal body temperature, observed changes in vulvar discharge (from cervical mucus) and collected first morning urine for hormonal testing. Additionally, they used home urine test sticks for LH. When the women observed mucus with estrogenic characteristics that are known to be associated with an approaching ovulation [14], or the stick indicated an LH peak (whichever came first), ultrasound to measure of follicular diameter was initiated. In each site all ultrasound measurements were conducted by a single physician. According to the choice of the study subject, the sonography was either transvaginal (58%) or transabdominal (42%), the modus was maintained for all further examinations of the same subject. An earlier analysis has shown no difference between both measurement approaches in respect to ovulation or mean follicular size in this dataset [13]. The ultrasound was performed every other day as long as the follicle size was less than 16 mm and thereafter on daily basis. In 45 (14%) cycles ovulation had already occurred at the time of the first ultrasound, or no ovulation could be demonstrated by ultrasound or hormonal markers. In the remaining 282 (86%) cycles, there was at least one measurement prior to ovulation, with ovulation documented by follicular collapse on subsequent measurement and confirmed by multiple hormonal measures. Most women contributed three cycles of observation, but there were different numbers of measurements per cycle and per woman. At each ultrasound investigation, the largest follicle was measured in two planes; we calculated the mean of these two measurements. For this analysis, we designated the day after the largest follicular diameter as the day of ovulation, which therefore corresponded to the first day of follicular collapse. In 40 cycles (14%) there was an interval of 2 days between the ultrasound showing the largest follicle and the ultrasound documenting follicular collapse (this could happen when the follicular diameter at the first measurement was below 16 mm and the woman was scheduled for the next measurement two days later or she missed her appointment on the following day). In these cases the following rule was applied to designate the day of ovulation: When the follicular diameter was ≥ 18 mm at the last pre-ovulatory ultrasound, the day immediately following was assumed to be the ovulation day. In the case when the follicle was <18 mm at first measurement, the second day following the measurement was assumed to be the day of ovulation. Additionally, there were 19 cycles where the ovulation day was imputed based on hormonal data. Alternate analyses including and excluding these cycles with an imputed day of ovulation were performed.

### Statistical analysis

The dataset had a hierarchical structure with three levels of clustering, each of which could potentially be a source of variability in follicular growth: center, woman and cycle. Given the assumption of linear growth (linear increase in diameter) on the days immediately preceding ovulation, different growth trajectories are defined by two parameters: slope (i.e. follicular growth rate in mm per day) and intercept, or maximum follicular diameter before ovulation. Differences in follicular growth patterns between centers, women or cycles of the same woman could be reflected in both of these parameters: different follicular growth rate and different maximum diameter.

To study the contribution of variability associated with each level to the total variability in the sample we used multi-level analysis [15]. Since a fully saturated model including sources of variability on all levels did not converge we proceeded with down-up modeling strategy: we investigated first the random intercept and slope in a joint model separately for each level. Then one of the random effects was removed at a time and the model was refitted to the data. Significance of the investigated random effect at the conventional level (p ≤ 0.05) was assessed on the basis of likelihood ratio test. The modeling strategy followed standard recommendations for mixed effects models [15]. After single random effects were identified a model including all significant random effects was fitted to the data. This model was further reduced by testing to determine which of the random effects could be excluded. The analysis was performed using PROC MIXED in SAS [16].

## Results

**Figure 1:**
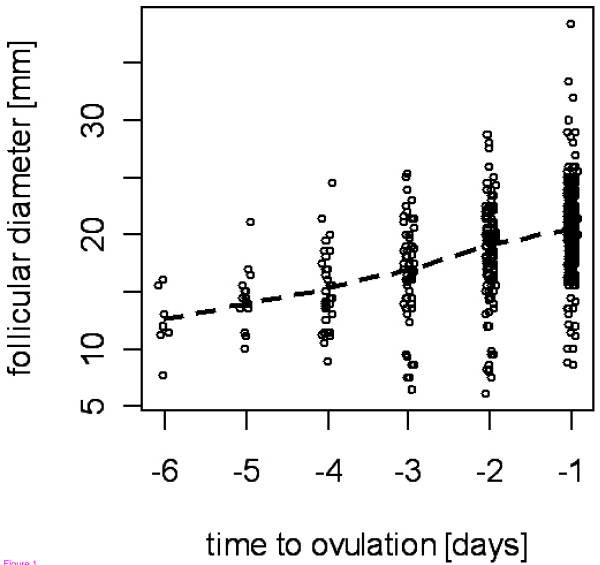
**Follicular diameter on days preceding ovulation**. Note: for a better visibility data are jittered on the time scale, scattered line shows a locally weighted regression for average trend.

The number of women per centre varied between 5 and 17 with most centres contributing measurements from 11 to 15 women. The number of recorded cycles varied per centre between 15 and 52 with most centres providing between 20 and 45 cycles. Most women contributed either 3 or 4 cycles with measurements and between 3 and 7 measurements overall. There were 152 cycles with 2 or more measurements (69 with more than two measurements) and 49 women had at least two cycles with at least 2 measurements in each cycle. There were 533 ultrasound measurements of follicular size before ovulation (with two diameters included in each measurement). Most of the measurements (520) preceded ovulation by 1 to 6 days. There was a large variability in follicular diameters observed on each day prior to ovulation (Fig. 1). The women investigated in different centres did not significantly differ in terms of age, body weight or height (data not shown).

**Figure 2:**
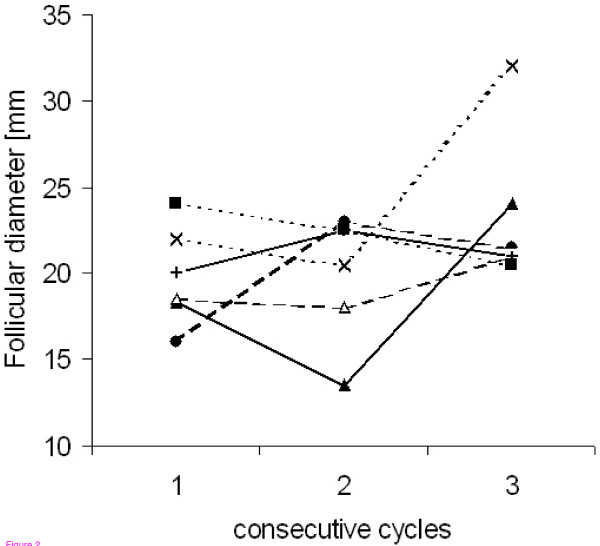
Maximum follicular diameter in consecutive cycles of 6 randomly selected women.

Analysis of the sources of variation in follicular growth showed no evidence for a center effect in relation to the follicular growth rate or maximum follicular diameter. There was a homogeneous follicular growth rate across women and there was no indication of differences between women in regard to maximum follicular diameter. Across cycles, the growth rate was likewise homogeneous, but there was a significant variation in maximum follicular diameter. Thus, follicular growth occurred at approximately the same rate, but the maximum size varied by the duration of the growth which varied from cycle to cycle. The variation in maximum follicle diameter in three consecutive cycles of 6 randomly selected women is illustrated in Fig. 2. The average growth rate estimated from the model was 1.68 mm/day and the average follicular diameter before ovulation was 20.13 mm.

**Figure 3:**
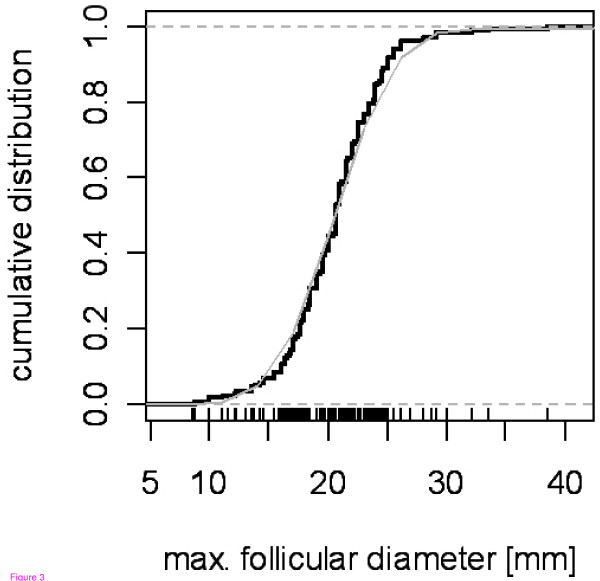
**Cumulative distribution of maximum follicular diameter prior to ovulation**. Note: black line – observed distribution, gray line – fitted normal distribution, N(20.48, 3.98).

There was a substantial variation in maximum follicular diameter before ovulation between cycles (Fig. 3). The distribution of follicular size before ovulation was close to a normal distribution with a mean value of 20.48 mm (based on observed data) and standard deviation of 3.98. In the observed data, about 40% of cycles had a follicular diameter <19 mm on the day prior to ovulation. Given that duration of the growth process determined follicular size at rupture it might be expected that bigger follicles would be observed in longer cycles. However, there was no such correlation (Spearman's rho = 0.02, p-value = 0.7).

## Discussion

We analyzed variability in the growth trajectories for ovarian follicles on 6 days preceding ovulation. The variability in maximum follicular size reached before ovulation was due to different time points of follicular rupture rather than different growth rates. While variation in maximum follicular size was also observed in previous studies, our new finding based on multilevel modeling, is that the variability in maximum follicular diameter is not due to systematic differences across women, but rather occurs on the cycle level.

Ultrasound evaluation of subfertile couples is often based on single cycles. While our analysis was restricted to women with no known fertility problems, our results suggest that the wide variation between cycles in follicular growth could result in misleading evaluation of follicular growth, when the assessment is based on single cycle.

Our findings confirm why prediction of time to ovulation based on follicular diameter is likely to result in a large uncertainty: same follicular diameter can be observed for different times to ovulation, because rupture may occur at different final follicular diameters. Our sample was restricted only to women who ovulated within seven days; if measurements had been made at earlier days prior to ovulation, some of them likely would have been large enough to result in even greater uncertainty about time to ovulation based on follicular size. Furthermore, additional follicular growth waves not leading to ovulation have been observed at other times of the cycle [17,18]. These would further increase the uncertainty in predicting time to ovulation from follicular diameter only. In this sample of women of normal fecundity follicular diameter of ovulation was not a specific characteristic of a given woman but rather varied randomly from cycle to cycle within each woman.

Further, we observed a substantial fraction of cycles with a relatively small follicle size. Since measurements were only made approximately every 24 hours, some of the small follicles may still grow before rupture, but given the estimated growth of 1.7 mm/day, for a substantial fraction of the follicles or cycles, ovulation will occur below 19 or 20 mm. Different follicular diameters at ovulation might be related to different reproductive outcomes. Maturation of the follicles has been shown to be related to outcomes in in-vitro fertilization in humans [19-24], with the size of the follicle being one of measures of follicle maturation. Different studies have used different cut off value for small follicles: <16 mm, <19 mm or even <20 mm. In a recent study of outcomes of ovulation induction and intrauterine insemination follicles smaller than 16 mm diameter at hCG administration resulted in lower rate of implantation [25]. Among women with known subfertility, sonographic abnormalities of follicular development were observed more frequently than among women of normal fertility [26,27]. Small follicles are also observed in women with polycystic ovary syndrome, a condition associated with infertility and early pregnancy loss [28]. Hilgers observed 8 abnormal pregnancies outcomes (miscarriage or ectopic pregnancies) among 13 clinical pregnancies resulting from natural intercourse in cycles with follicles with diameter <19 mm before ovulation [29]. However, since his sample included mainly subfertile women, it is not clear whether a small follicular size is related to adverse pregnancy outcomes in women of normal fertility. Follicular size might be a cause or a symptom of an underlying condition. We found that follicles smaller than 19 mm occur relatively frequent in cycles of women of normal fecundity. It is possible that small follicular size may be related to fertilization or implantation failure and might help explain that the average per cycle probability of getting pregnant is only 30% across multiple studies [30,31].

There are several limitations to our analysis. The original study did not conduct a formal evaluation of the reliability of ultrasound measurements conducted in this study. However, all ultrasound measurements within each center were done by a single physician, so the absence of the center effects suggests that no systematic bias was introduced by measurements conducted by different physicians. We believe that this study represents what may be expected from careful clinical collection of ultrasound data on follicular growth. Although we did not find any evidence for differences in follicular size between women, this study did not include subfertile women, by design. Inclusion of subfertile women may have resulted in differences between women, and possibly more variation in growth trajectories or final diameters. Another limitation is that the ultrasound measurements were most often performed on one to two days before ovulation, and many cycles contained only one or two measurements. While the methodology of mixed models allows for inclusion of incomplete information, a large amount of missing data can still result in bias of the analysis, especially with regard to the detection of variation. This might have resulted in unrecognized variability in the rate of follicular growth across women or centers. Since the dataset included only 8 centers in the analysis, the chance to detect a random effect on this level was limited. The centers were all located in Europe and we cannot exclude the possibility that there are systematic differences between non-represented ethnic groups.

## Conclusion

We demonstrated that main variability in follicular diameter reached before ovulation is across cycles and depends on the duration of the growth process rather than on different growth rates. We did not detect any significant differences in follicular growth across the studied populations and across women. We also found that follicular size before ovulation is often below 19 mm, a size that has been reported to be associated with miscarriages in subfecund women. It is not clear if this is of importance for women with no known fertility problems as in our study. Further research in other populations is needed to confirm these results and to investigate potential causes or correlates of the variation in maximum follicle size reached before ovulation across cycles, and the potential association with reproductive outcomes. Further research should also include a joint modeling of ultrasound and hormonal measurements.

## Competing interests

The authors declare that they have no competing interests.

## Authors' contributions

RTM developed the research question, conducted the analysis and wrote the draft of the manuscript. JBS contributed to writing the final version. RE performed the original study and provided comments on the manuscript. All authors read and approved the final manuscript.
